# A structural analysis of the AAA+ domains in *Saccharomyces cerevisiae* cytoplasmic dynein

**DOI:** 10.1016/j.jsb.2014.03.019

**Published:** 2014-06

**Authors:** Emma S. Gleave, Helgo Schmidt, Andrew P. Carter

**Affiliations:** MRC Laboratory of Molecular Biology, Francis Crick Ave, Cambridge CB2 0QH, UK

**Keywords:** Microtubule, Cytoskeleton, Motor protein, Dynein, ATPases Associated with diverse cellular Activities (AAA+), Coiled-coil

## Abstract

Dyneins are large protein complexes that act as microtubule based molecular motors. The dynein heavy chain contains a motor domain which is a member of the AAA+ protein family (ATPases Associated with diverse cellular Activities). Proteins of the AAA+ family show a diverse range of functionalities, but share a related core AAA+ domain, which often assembles into hexameric rings. Dynein is unusual because it has all six AAA+ domains linked together, in one long polypeptide. The dynein motor domain generates movement by coupling ATP driven conformational changes in the AAA+ ring to the swing of a motile element called the linker. Dynein binds to its microtubule track via a long antiparallel coiled-coil stalk that emanates from the AAA+ ring. Recently the first high resolution structures of the dynein motor domain were published. Here we provide a detailed structural analysis of the six AAA+ domains using our *Saccharomycescerevisiae* crystal structure. We describe how structural similarities in the dynein AAA+ domains suggest they share a common evolutionary origin. We analyse how the different AAA+ domains have diverged from each other. We discuss how this is related to the function of dynein as a motor protein and how the AAA+ domains of dynein compare to those of other AAA+ proteins.

## 1 Introduction

Dyneins are a family of motor proteins that move to the minus end of microtubules. They are all multiprotein complexes assembled around a 4000+ amino acid long “heavy chain.” The dynein subfamilies are classified based on their heavy chain (Gibbons, 1995; Wickstead and Gull, 2007). The cytoplasmic dynein (cytoplasmic dynein-1) subfamily are transporters. They carry a large range of cargos, from individual mRNAs to whole organelles (Vallee et al., 2004) and also play numerous roles in mitosis (Vaughan, 2012). The intraflagella transport dynein (IFT dynein or cytoplasmic dynein-2) subfamily also transports cargos, but predominantly along the axoneme (Lye et al., 1987). The remaining subfamilies are all axonemal dyneins which are responsible for the beating movement of cilia and flagella (DiBella and King, 2001). The axonemal dyneins are divided into the outer-arm dyneins (α/β and γ) and the more numerous inner-arm dyneins based on their position within the axoneme (Piperno et al., 1990).

All dynein heavy chains share a conserved motor domain that is related to the AAA+ (ATPases Associated with diverse cellular Activities) family. Proteins of the AAA+ family have many diverse functions (Erzberger and Berger, 2006). Some, such as the proteasome lid (Baumeister and Lupas, 1997) and ClpX (Siddiqui et al., 2004), unfold proteins so they can be fed into protein degradation machines. Others are involved with disassembling large protein complexes: katanin severs microtubules (McNally and Vale, 1993) and Vps4 takes apart ESCRT filaments (Babst et al., 1998). There are also remodelers, which change the conformation of other proteins: PspF (Buck et al., 2000) and NtrC (Lee et al., 2003) work on the sigma factor σ_54_ involved in transcription, while the clamp loader opens the PCNA ring so it can be loaded onto DNA (Neuwald, 2005). Other AAA+ family members are helicases that unwind DNA or RNA (Singleton et al., 2007).

**Fig.1:**
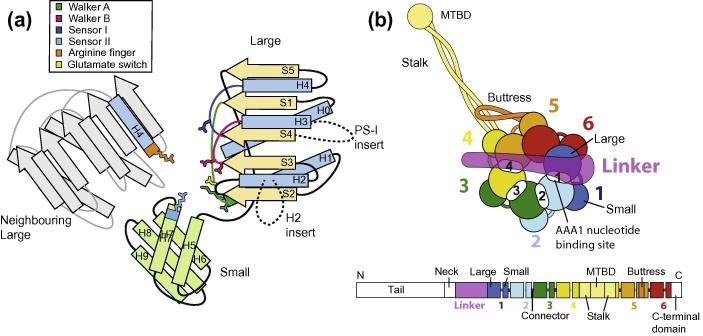
The dynein motor domain is composed of AAA+ domains. (a) Schematic diagram showing the key features of a AAA+ domain. The large subdomain contains a central β-sheet (yellow) with flanking helices (blue) and the small subdomain often contains a bundle of α-helices (green). The nucleotide binding pocket is located at the cleft between a AAA+ domain (colour) and the neighbouring large AAA+ subdomain (grey). Key residues involved in nucleotide binding and hydrolysis are shown in cartoon form and coloured according to the key. The large subdomain contributes the Walker-A, the Walker-B, sensor I and the glutamate switch motifs, the small subdomain has the sensor II motif and the neighbouring large subdomain provides the arginine finger. Some of dynein’s AAA+ large subdomains contain inserts, notably a pre-sensor-I (PS-I) and a helix 2 (H2) insert as indicated by the dashed lines. (b) Schematic representations of the dynein motor domain and the heavy chain gene. The upper cartoon depicts the main features of the motor domain. The colour-coded AAA+ subdomains are represented as large (large AAA+ subdomain) and small (small AAA+ subdomain) circles. The nucleotide binding sites are shown as white ovals labelled 1–4. The N-terminal linker (purple) spans across the AAA+ ring. The stalk and the buttress are coiled-coil extensions of AAA4S and AAA5S, respectively. The colour-coded dynein heavy chain gene organisation is shown underneath the cartoon (connector peptides are shown in pink).

A common feature of AAA+ proteins is that they contain a number of conserved AAA+ domains (Fig. 1). Each of these consists of an N-terminal “large” subdomain (also called the α/β subdomain) and a C-terminal “small” subdomain (also called a α-domain or the C-domain (Ammelburg et al., 2006)). The large subdomain is structurally more conserved and is made up of a central β-sheet and flanking α-helices (Fig. 1a). The small subdomain is more variable in structure, but often contains a bundle of α-helices. The ATP adenine base binds in a pocket between the large and small subdomains. The large subdomain contributes the Walker-A motif, that binds the ATP α and β phosphates and the Walker-B and sensor I motifs that coordinate the water molecule required for ATP hydrolysis (Wendler et al., 2012). In some family members, the small subdomains contribute an arginine residue (sensor II) to the ATP binding site. The neighbouring AAA+ domain contains another arginine residue (arginine finger), whose movement into the ATP binding site is required for hydrolysis (Karata et al., 1999).

Another feature of AAA+ proteins is that the AAA+ domains are usually arranged in rings. In most proteins, the ring is made up of an oligomer of six individual proteins. However, exceptions do exist including incomplete rings and rings with more than six domains (Lee et al., 2003; Mizuno et al., 2013; Simonetta et al., 2009). Dyneins, together with the ribosome biogenesis factor midasin/Rea1 (Garbarino and Gibbons, 2002), are unusual in that all six of their AAA+ domains are arranged in tandem in one long polypeptide (Fig. 1b). In order to function as a motor, dyneins also have an N-terminal linker domain that acts as the force producing motile element (Fig. 1b). It swings across the AAA+ ring in response to nucleotide binding and hydrolysis (Burgess et al., 2003). All dyneins also have a long coiled-coil projection emerging from the AAA+ ring called the stalk (Gee et al., 1997), which has the microtubule binding domain (MTBD) at its tip (Fig. 1b). The buttress (or strut) is a shorter coiled-coil extension also emanating from the AAA+ ring, which supports the base of the stalk (Carter et al., 2011).

Recently, crystal structures of the cytoplasmic dynein-1 motor domain were determined from both *Saccharomyces cerevisiae* and *Dictyostelium discoideum* (Carter et al., 2011; Kon et al., 2011, 2012; Schmidt et al., 2012). These structures revealed a wealth of information about how the motor generates force. Here we perform a structural analysis of the *S. cerevisiae* motor domain to explore how its six AAA+ domains are related to each other and how they have specialised to perform different functions. We will discuss what this tells us about the evolution and function of dynein.

## 2 Methods

### 2.1 Protein structure visualisation

All protein structure images were generated using PyMOL (version 1.5.0.5) (Schrodinger, 2010). Structural alignments were performed using the built-in cealign command of PyMOL.

### 2.2 Domain boundaries

The domain boundaries (Table 1) were defined based on the secondary structure prediction (Carter et al., 2011) and confirmed by the position of residues in a high resolution dynein structure (PDB ID: 4AKI). The domains were subjected to structural alignment in PyMOL with the cealign command. This revealed core large and small subdomains which showed high structural conservation between the dynein domains and other AAA+ proteins. In addition to the core features, inserts and connector peptides were defined. The inserts and connectors peptides varied between dynein’s domains.

### 2.3 Sequence alignments

Dynein sequences from a range of families and species were identified by performing a protein BLAST (blastp) search of NCBI using the *S. cerevisiae* dynein motor domain as a search model. Sequence alignments used ClustalX (Larkin et al., 2007) and were analysed with BioEdit (Hall, 1999). Alignments were annotated using Jalview (Waterhouse et al., 2009).

### 2.4 Small subdomain analysis

Analysis of the sequence and structural relationships between small subdomains was performed using the MultiSeq plugin of VMD (Roberts et al., 2006). After a STAMP structural alignment a QH structural phylogenetic tree was generated. The tree in Fig. 4 was generated using the following proteins (PDB ID and residue numbers used are given): Dyn-AAA1S (4AKI: 1926–2005), Dyn-AAA2S (4AKI: 2222–2345), Dyn-AAA3S (4AKI: 2559–2680), Dyn-AAA4S (4AKI: 2917–3009 + 3316–3355), Dyn5 (4AKI: 3519–3537 + 3617–3710), Dyn6 (4AKI: 3898–4015), MoxR (3NBX: 177–307), BchI (1GP8: 269–350 and 219–317 from a symmetry related monomer), NtrC (3M0E: 321–384), PspF (2C9C: 191–258), ClpA-D2 (1R6B: 663–724), ClpB-D2 (1QVR: 769–830), HslU (1G41: 351–431), ClpX (3HTE: 332–403), LonA (3M6A: 493–582), Proteasome (4COV: 377–456), PAN (3H4M: 338–418), FtsH (1LV7: 322–398), p97-D1 (3CF1: 369–434 + 445–458), p97-D2 (3CF1: 646–744), NSF (1NSF: 667–735), Vps4 (2QPA: 296–413), Spastin (3VFD: 507–593), RuvBL12 (2XSZ: 274–362), ClpB-D1 (1QVR: 330–395 + 521–534), ClpA-D1 (1R6B: 350–434), ClpC-D1 (3J3R), ClampLoader (1JR3: 175–241), RuvB (1HQC: 165–239), DnaA (1L8Q: 265–302), DnaA2 (2Z4R), Cdc6 (2QBY: 206–292), Apaf1 (3J2T: 280–350), Ced4 (3LQQ: 291–364).

## 3 Results and discussion

### 3.1 The core AAA+ domains of dynein are structurally similar to each other

The dynein motor domain (Fig. 1b) contains six AAA+ domains referred to as AAA1–AAA6. AAA1 contains the main site of ATP hydrolysis in dynein (Kon et al., 2004). Domains AAA2–4 can also bind nucleotide. Domains AAA5 and AAA6 have lost their nucleotide binding sites. All the AAA+ domains in dynein contain both a large (e.g. AAA1L) and a small (e.g. AAA1S) subdomain. The regions that link one AAA+ domain to the next are referred to as connectors. The region following AAA6 is called the C-terminal domain. The first part of this C-terminal domain is present in all dyneins (Carter et al., 2011; Numata et al., 2011) and stretches back towards AAA5. The second part contains an additional six α-helices and a β-barrel (Kon et al., 2012) and is absent in some dyneins, including *S. cerevisiae*. Table 1 lists the main domain boundaries in the cytoplasmic dyneins that have had their structures solved.

**Fig.2:**
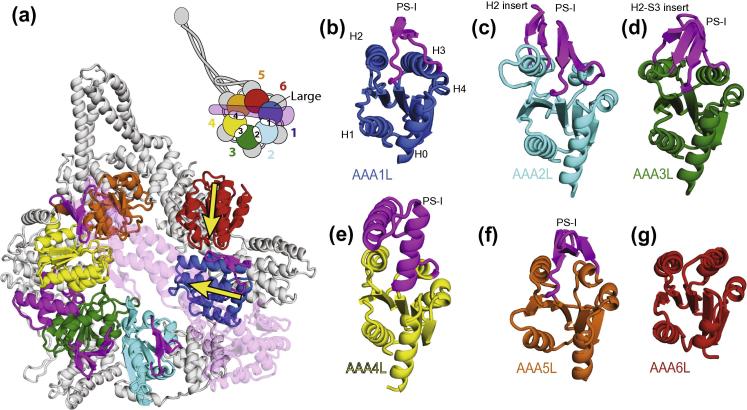
Dynein contains six large AAA+ subdomains. (a) A ribbon representation of the *S. cerevisiae* motor domain (PDB-ID: 4AKG). Large subdomains are coloured according to the schematic representation, from blue to red. The linker sits on top of the ring and has been made transparent for clarity. Each large subdomain contains a central β-sheet that is flanked by α-helices. Inserts (PS-I and/or H2) are shown in magenta protruding onto the linker face of the ring. The direction of the central β-sheets of AAA1L and AAA6L are indicated by large yellow arrows. (b–g) The individual AAA+ large subdomains are shown in the same orientation. All large subdomains share a common core structure. Helices 1–6 have been labelled in (b). Inserts are coloured in magenta and project from the top of the large subdomain.

The dynein AAA+ domains contain a typical AAA+ fold. The large subdomains contain a central five-stranded parallel β-sheet (strands S1–S5), flanked on one side by helices H0 and H1 and on the other by helices H2–H4 (Fig. 1a). The cores of the AAA+ large subdomains of dynein are structurally similar to each other (Fig. 2). The largest differences between them are the inserts (highlighted in magenta in Fig. 2), which will be discussed further below.

In five of the AAA+ large subdomains (AAA1–5), the strands of the β-sheet point approximately towards the centre of the ring. However, the AAA6 large subdomain has rotated so that its β-sheet is orientated almost tangentially to the ring (Fig. 2a yellow arrows). This can probably occur because of the loss of the nucleotide binding site in AAA6. As a result, the AAA6 large subdomain interacts with its neighbouring AAA1 domain differently from the other AAA+ domains. A comparison between the *S. cerevisiae* and *D. discoideum* structures shows flexibility in this interface (Carter, 2013), suggesting it might be important for accommodation of the nucleotide dependent changes that occur elsewhere in the dynein ring as nucleotides are bound and hydrolysed.

**Fig.3:**
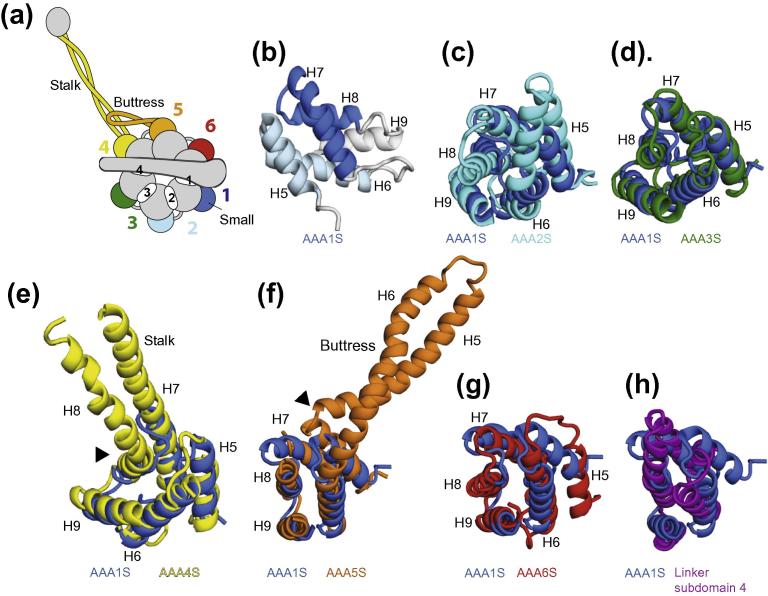
The small AAA+ subdomains of dynein. (a) Schematic representation highlighting the positions of the small subdomains on the outside of the ring. (b) Small subdomain of AAA1 coloured in different shades of blue to highlight the structure which consists of a five helix bundle containing two α-hairpins. Helices 5 and 6 form the first hairpin (light blue), the second hairpin is formed by H7 and H8 (dark blue). H9 is shown in grey. (c–g) An alignment of small domains AAA2–6 (cyan–red) with AAA1 (blue) shows that all small subdomains share the same topology. Coiled-coil extensions in the small subdomain are found in AAA4S (e) and AAA5S (f) and are named the stalk and buttress, respectively. The extended helices contain kinks (black arrows) to allow them to join onto the core coiled-coil structure. (h) The linker contains a five helix bundle that aligns well with AAA1S.

The small AAA+ subdomains of dynein all contain a core bundle of five alpha helices (H5–H9) (Figs. 1a, 3b). The topology of the helices is the same for all subdomains and the core regions align well with each other. This is shown in Fig. 3c–g by aligning each small subdomain onto that of AAA1S. In common with many other AAA+ small subdomains (Ammelburg et al., 2006; Alva et al., 2007), the first four helices (H5–H8) in the dynein small subdomains form a superhelical bundle of two helical hairpins. The first helical hairpin (H5- and H6-light blue in Fig. 3b) adopts a similar conformation as the second pair of helices (H7- and H8-dark blue in Fig. 3b). Both pairs of helices cross each other at a similar angle and can be aligned on top of each other. The position of the fifth helix (H9) is the same in all the dynein small subdomains.

While there are some similarities between small subdomains in different AAA+ proteins they tend to be the most variable part of the AAA+ domain (Ammelburg et al., 2006). Given this variability, the similar fold of all six dynein small subdomains is striking. It suggests two possibilities. One is that the six dynein AAA+ domains are directly related to each other and arose by duplication of an ancestral AAA+. The other is that the domains have independently evolved a fifth helix in a similar position perhaps for a functional reason.

**Fig.4:**
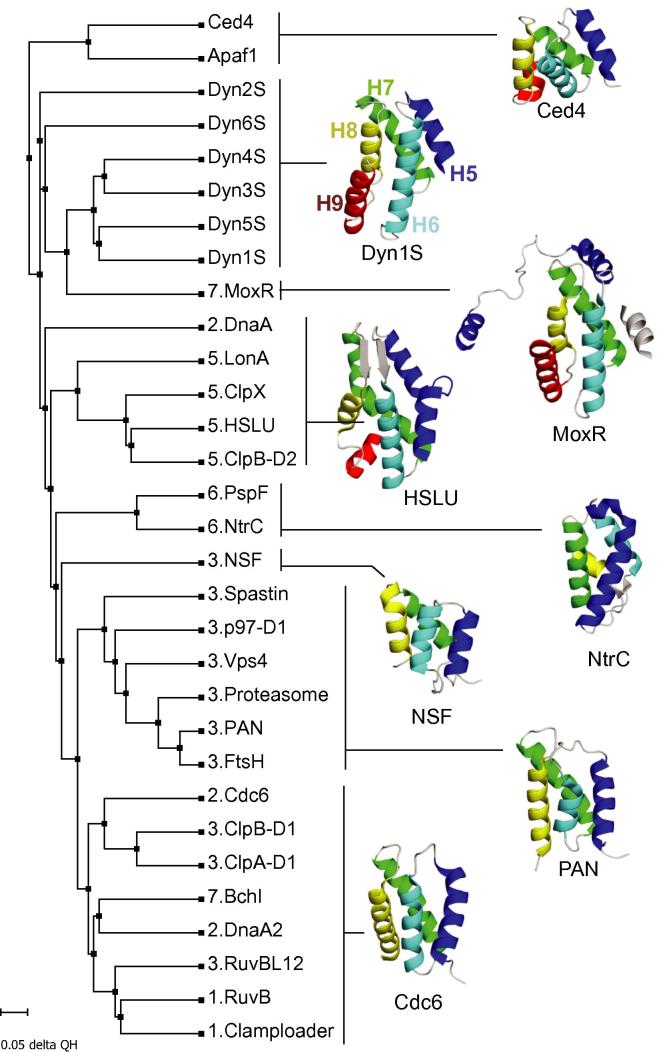
A structural phylogenetic tree shows the relationships between small subdomains. The phylogenetic tree was prepared using the MultiSeq function of VMD. The number preceding the protein name indicates the clade to which the protein belongs (Erzberger and Berger, 2006). A representative small subdomain structure for each cluster of proteins is shown in ribbon form. The small subdomains of the proteins are aligned to the first four helices (H5–H8) of Dyn1S. The small subdomain helices are coloured as follows: H1-blue, H2-cyan, H3-green, H4-yellow and H5-red. The MoxR image contains an additional helix (grey) from a symmetry related molecule which packs against the small subdomain.

In order to obtain an idea of how closely related the dynein AAA+ small subdomains are we performed a structural based alignment of a selection of small subdomains from AAA+ proteins from different clades (Erzberger and Berger, 2006). We used the program Multiseq implemented in the VMD software (Roberts et al., 2006) to perform the alignments of the structurally conserved regions and to generate a structural phylogenetic tree. In general, the small subdomains belonging to different clades are structurally similar and cluster together (Fig. 4), although there are some exceptions. Among the *S. cerevisiae* dynein small subdomains, AAA1S, AAA3S, AAA4S and AAA5S cluster together and therefore are the most similar. They also cluster together if only the first four helices (H5–H8) are used in the alignment (data not shown). The small subdomains of AAA2 and AAA6 have diverged more. Their clustering with the other AAA+ domains depends on the presence of the fifth helix (H9).

The evolution of dynein pre-dates the last eukaryotic common ancestor which already possessed differentiated cytoplasmic, IFT and axonemal dynein families (Wilkes et al., 2008). The structural phylogenetic tree provides some support for the idea that this evolution of dynein involved the repeated duplication of an ancestral AAA+ domain. It has previously been proposed (Asai and Koonce, 2001), that domains AAA1–4 and AAA5–6 arose independently and then joined together. This is possible, but if it occurred then both sections would still have to have been related to a common ancestral AAA+ domain. Interestingly, the last subdomain of the dynein linker (subdomain 4), just before AAA1, contains a bundle of five helices that shares the same topology as the six dynein AAA+ small subdomains (Fig. 3h). While the structural alignment is less good (6 Å RMSD vs 3.5–4 Å for the other small subdomains), the similarity in helix topology in the linker subdomain suggests it may be a remnant of a 7th ancestral AAA+ domain that arose during the duplication of the AAA+ domains.

### 3.2 The AAA+ domains of dynein contain different specialisations

While the core structure of the six dynein AAA+ domains is conserved, they have all diversified from each other. The most obvious example of this is the nucleotide binding sites. Other AAA+ proteins have multiple nucleotide binding sites that hydrolyse ATP and contribute to function (Lupas and Martin, 2002). In contrast, only the first AAA+ domain (AAA1) is conserved between all dyneins. AAA2 has lost all ability to hydrolyse nucleotide and uses ATP in a structural role to hold the AAA2–AAA3 interface tightly closed (Schmidt et al., 2012). AAA3 retains the residues for ATP hydrolysis in cytoplasmic dyneins but not in axonemal or IFT dyneins. AAA4 binds nucleotide in at least some dyneins, while AAA5 and AAA6 have lost all binding activity. The specialisation of the nucleotide binding sites has been reviewed extensively (Carter, 2013; Roberts et al., 2013) and will not be further discussed here. Instead, we will consider other specialisations including: inserts into the core AAA+ large subdomains, additions to the small subdomains and the connectors between the AAA+ domains.

### 3.3 Large subdomains contain different combinations of inserts

Many of the AAA+ family proteins have inserts in the conserved core of the large subdomains that play direct roles in their function (Erzberger and Berger, 2006). The two most common inserts are the pre-sensor-I insert (PS-I), found between H3 and S4 and the H2 insert, found in the middle of H2 (Iyer et al., 2004) (Fig. 1a). The first five (AAA1–AAA5) AAA+ large subdomains of dynein have PS-I and/or H2 inserts, each of which is structurally different. Interestingly, no inserts are present in the AAA6 large subdomain. In order to address the function of the different large subdomain inserts we have analysed their conservation among dyneins. In particular we have looked at surface exposed residues, which are typically conserved only if they are involved in protein interactions. Sites of interaction that are found in all dyneins are likely to be fundamental to the motor mechanism, whereas those that are only found in one class of dyneins may have more specific functions.

#### 3.3.1 AAA1

**Fig.5:**
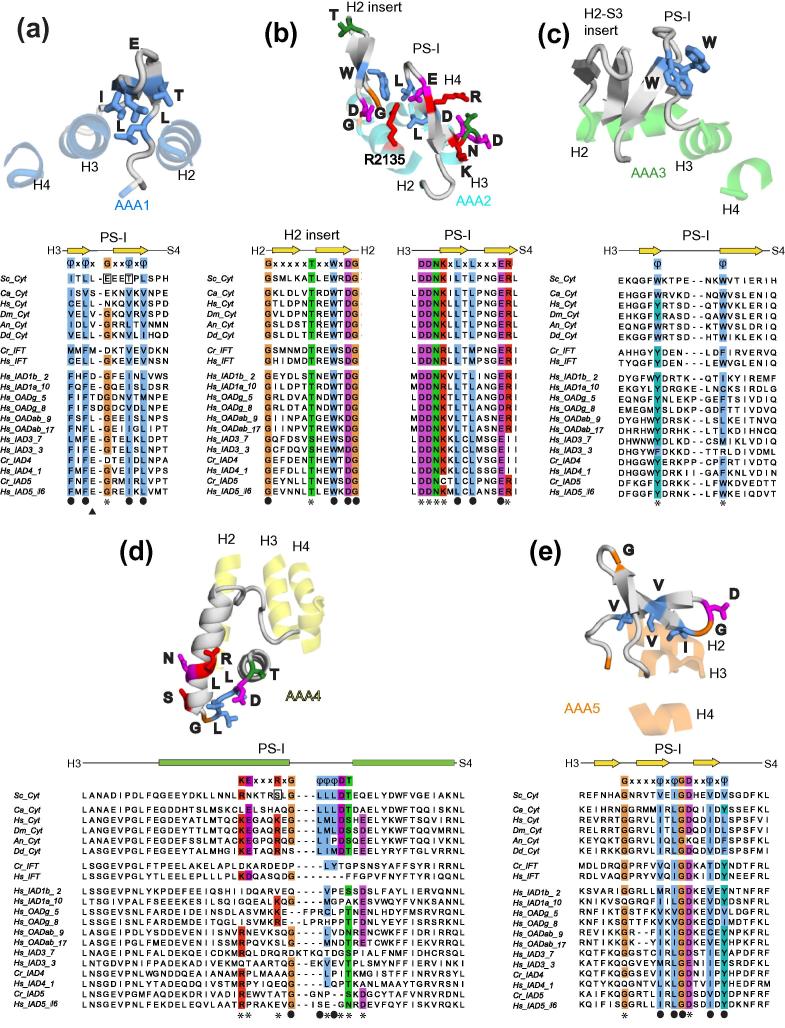
Consensus sequences in the PS-1/H2 inserts of the dynein AAA+ large subdomains. (a–e) The inserts and helices H2, H3 and H4 of AAA+ domains AAA1–5 are shown in ribbon representation above alignments of the insert sequences from different dyneins. Conserved resdiues in the inserts are highlighted. Amino-acid residues are colour-coded according to their chemical properties: blue: hydrophobic, green: hydrophilic, red:basic and magenta: acidic. Glycine residues are highlighted in orange and tyrosine residues in cyan. Surface exposed conserved residues are highlighted with an asterisk and structural residues with a solid black circle. Amino-acid residues of the AAA1L–AAA5L insert consensus sequences of the *S. cerevisiae* dynein motor crystal structure are shown as stick representation. Cyt: cytoplasmic dynein, IFT: intraflagellar transport dynein, IAD: axonemal inner-arm dynein, OAD: axonemal outer-arm dynein.

AAA1 contains a β-hairpin PS-I insert which folds back over the large subdomain (Figs. 2b and 5a). The insert shows weak electron density in the *S. cerevisiae* dynein motor domain crystal structure and is partially disordered in the *D. discoideum* dynein crystal structure. A sequence alignment of different dyneins (Fig. 5a, lower panel) shows that the insert contains a conserved φxφxGxxφxφ (where φ is a hydrophobic residue) motif. The hydrophobic residues in the motif are structural and cover the cleft between H2 and H3. The surface exposed residues in AAA1 PS-I are not conserved in cytoplasmic dyneins. In some axonemal dynein families (inner arm dynein families IAD3, IAD4 and IAD5), there is a conserved glutamate (E) residue in the 4th position of the motif (arrow in Fig. 5a). This residue lies on the exposed face of the β-hairpin and lies close to the AAA6 domain. It is positioned to interact with the AAA6 domain when it moves to accommodate conformational changes in the AAA+ ring (Carter, 2013).

#### 3.3.2 AAA2

The AAA2 large subdomain contains both a PS-I and H2 insert (Fig. 2c). These inserts play a direct role in bending the dynein linker so it can swing across the AAA+ ring. When ATP binds at AAA1, the site closes and brings the AAA2 inserts into contact with the linker (Kon et al., 2012; Roberts et al., 2012; Schmidt et al., 2012). Consistent with this key role, the residues in these two inserts are highly conserved among all dynein families. The H2 insert contains the motif GxxxxxT/SxxWxDG and the PS-I insert contains the motif DDNKxLxLxxxER (Fig. 5b). Some of these residues have purely structural roles. The flanking glycine residues in the H2 insert allow it to emerge smoothly from the H2 helix. The tryptophan (W) in H2 and two leucine (L) residues in PS-I form a hydrophobic core between the H2 and PS-I insert (Fig. 5b). The aspartate (D) in H2 and the glutamate (E) in PS-I form a network of salt bridges with an arginine (R2135) from helix 2. These residues hold the inserts so rigidly that they were identifiable even in a low resolution crystal structure of the dynein motor domain (Carter et al., 2011). The inserts also contain a number of highly conserved surface exposed residues that are candidates for interacting with the linker domain. These are the threonine or serine (T/S) on H2, the DDNK motif at the base of PS-I and to a lesser extent the arginine (R) at the other end of PS-I.

The combination of H2 and PS-I inserts is seen in other AAA+ proteins such as NtrC, PspF, the Mg-chelatase BchI and MoxR (Erzberger and Berger, 2006). In PspF and NtrC (Buck et al., 2000; Lee et al., 2003) the inserts contact the σ_54_ bound RNA polymerase complex and “remodel” it to open up the DNA and start transcription (Studholme and Dixon, 2003). This may be analogous to the role the AAA2 inserts play in “remodelling”/bending the dynein linker (Cho and Vale, 2012). A key difference between dynein and NtrC/PspF is the flexibility of the H2 insert and how it responds to nucleotide. In PspF the conformation of the H2 insert (referred to as Loop-1) is coupled, via interactions involving a conserved asparagine residue (the glutamate switch (Zhang and Wigley, 2008)), to the ATP binding site. In the presence of ATP, the H2 insert (referred to as Loop-1) is ordered and can bind σ_54_. In the presence of ADP, the glutamate switch asparagine changes its interaction with the Walker-B glutamate (Rappas et al., 2006). This leads to the H2 insert folding back on itself and burying the GAFTGA motif at its end so that σ_54_ is released (Buck et al., 2000). In dynein the nucleotide binding site of AAA2 appears to harbour a purely structural, non-hydrolysable and non-exchangeable ATP (Carter, 2013; Carter et al., 2011; Schmidt et al., 2012) and lacks the glutamate switch residues. In addition, the AAA2 H2 insert appears to be a much more rigid structure than the PspF H2. Therefore in dynein the H2 and PS-I remodel the linker but are not directly involved in signalling back to the ATP site.

#### 3.3.3 AAA3

The AAA3 large subdomain contains two inserts in the core domain. One is a PS-I β-hairpin between H3 and S4 and the other is another β-hairpin positioned between H2 and S3 (Figs. 2d and 5c). The surface exposed residues in these inserts are poorly conserved among different dyneins, with the exception of two hydrophobic residues in PS-I (marked with an asterisk in Fig. 5c). In many dyneins the residues at these positions are aromatics, typically tryptophan (W) or tyrosine (Y), or hydrophobic residues. In *S. cerevisiae* dynein both positions are occupied by tryptophans that form hydrophobic contacts with AAA2. These interactions, together with a network of salt bridges to a “structural” ATP residue (Carter, 2013; Schmidt et al., 2012) help stabilise the AAA2/AAA3 interface to keep it tightly closed.

#### 3.3.4 AAA4

The AAA4 large subdomain contains a single insert in the PS-I position. However, unlike the other PS-I inserts, it consists of a pair of α-helices (Fig. 5d). The helices are joined by a loop which varies in length among IFT and inner arm axonemal dyneins (Fig. 5d). The insert’s sequence is not conserved among all dyneins, but does show a strong degree of surface conservation among cytoplasmic dyneins. The end of the first helix and the loop contains the residues KExxxRxGφφφDS/T (Fig. 5d). The KExxxR motif residues are all exposed and are conserved in most cytoplasmic dyneins. The second hydrophobic (φ) residue and the DS/T in the φφφDS/T motif are even more conserved and are also exposed. This strongly suggests that the AAA4 PS-I insert forms a functionally important interaction site in cytoplasmic dyneins. Possible candidates for binding to this region include part of Lis1, which is known to bind to a nearby patch of residues on the AAA3/AAA4 connector (Huang et al., 2012). Alternatively it could be another dynein regulatory factor that affects motor activity, such as dynactin (Kardon and Vale, 2009), or even the other motor domain in the dynein dimer. In support of the last suggestion, the PS-I insert was involved in a packing contact against a symmetry related dynein molecule in the *S. cerevisiae* dynein crystal structure (Schmidt et al., 2012).

#### 3.3.5 AAA5

The AAA5 large subdomain contains an insert in the PS-I position, which consists of three β-strands (Figs. 2f and 5e). The insert contains a conserved GxxxxφxφGDxxφxφ motif in all dynein families (Fig. 5e). The two glycines contribute to the tight turns between the three β-strands. The hydrophobic residues (φ) are found in the second and third β-strands and interact with helices H2 and H3 in AAA5. These second and third β-strands of the AAA5 PS-I align well with the two β-strands in PS-I of AAA1 and make similar contacts with the core of the AAA+ large subdomain. In the *S. cerevisiae* dynein structure the cleft between AAA4 and AAA5 is closed and the conserved aspartate (D) residue in the AAA5 PS-I insert is involved in making contacts with the AAA4 domain (Schmidt et al., 2012). The whole PS-I insert is disordered in the *D. discoideum* motor domain structure in which the cleft between the AAA4/5 domains has opened up (Kon et al., 2012). The AAA5 PS-I insert is next to a docking site, for the linker onto the AAA+ ring (Schmidt et al., 2012), but does not directly contribute to it. Instead its function may be to contribute to stabilizing the closure of the AAA4/5 cleft, which is the AAA4 nucleotide binding site (Carter, 2013; Kon and Kurisu, 2013).

### 3.4 Divergence of some small subdomains is related to function

The dynein AAA+ small subdomains have also diversified relative to each other. The small subdomain of AAA2 is longer than that of AAA1 (Fig. 3c). Helices H6–H8 are approximately 1.5× the length. The H6 helix is split into two with a short non-helical section between them (Fig. 3c). The small subdomain of AAA2 sticks out of the AAA+ ring to produce a feature that was observed in early negative stain structures of dynein (Burgess et al., 2004). In contrast, AAA3S is the most similar in overall fold to the AAA1S, with no obvious specialisations (Fig. 3d).

The specialisations of the AAA4 and AAA5 small subdomains were crucial to the development of dynein as a motor protein that can move along microtubules. In both cases this involved extension of a pair of helices. In AAA4S the third and fourth helices (H7 and H8) were elongated (Fig. 3e) to form the stalk. In AAA5S the first and second helices (H5 and H6) were elongated (Fig. 3f) to form a coiled-coil hairpin, called the buttress (Carter et al., 2011). The buttress supports the base of the stalk and is likely to communicate conformational changes in the AAA+ ring to the stalk and subsequently on to the tip of the stalk (Carter, 2013; Roberts et al., 2013). The dynein small subdomains are arranged radially around the AAA+ ring (Fig. 3a), which means that in order for the stalk and buttress to interact they have to bend to meet each other. This is achieved by a kink in AAA4S H8 that directs the stalk towards the buttress (Fig. 3e arrowhead) and a sharp 90° bend in AAA5S helix H5 that directs the buttress back towards the stalk (Fig. 3f arrowhead).

### 3.5 Structurally diverse connector sections link neighbouring AAA+ domains

**Fig.6:**
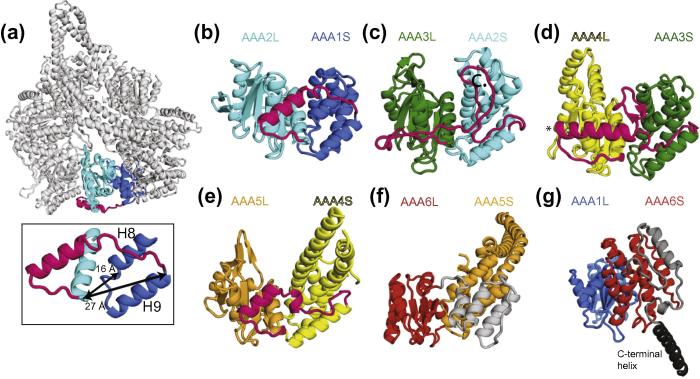
Dynein contains connectors between AAA+ domains. (a) Ribbon representation shows the position of AAA1S (dark blue) linked to AAA2L (cyan) via a connector (magenta) which wraps around the outside of the ring. The box inset shows that the distance from H8 of AAA1S to H0 ofAAA2L (16 Å) is shorter than the distance from H9 of AAA1S to H0 of AAA2L (27 Å). (b–f) Connector peptides (magenta b–e, grey in f) join each small subdomain to the next large subdomain. The connectors in the dynein motor domain have diversified extensively and can consist of helices, loops and extended peptide regions. The position of the conserved region in the AAA3S–AAA4L connector that is involved with Lis1 binding is highlighted with an asterisk in 6d. (g) The AAA6 small subdomain is followed by a connector region (shown in grey) that packs against the small subdomain. This is followed by a C-terminal helix (dark grey) that runs behind the AAA5S subdomain.

The dynein AAA+ domains are joined together by connector sections that join one small subdomain to the next large subdomain. This distance is actually larger in dynein than it would be in other AAA+ proteins that only have four helices in their small subdomain. The distance from the end of the 5th helix (H9) of one small subdomain to the first helix (H0) of the neighbouring large subdomain is an average of 28 Å (Fig. 6a. box). By contrast the distance between the 4th helix (H8) to the first helix (H0) of the next AAA+ large subdomain is on average 16 Å. Therefore, the presence of a fifth helix in the dynein small subdomains means an increased length of protein sequence is required to connect its AAA+ domains together. Midasin/Rea1, the other protein with all six AAA+ domains in one polypeptide, is also predicted to have a five helix small subdomain. There may be some advantage to both proteins to having extra sequence in their connector regions.

The connectors themselves have diverged extensively from each other (Fig. 6b–f). However, they are mainly well ordered structures that pack in between the small and neighbouring large subdomains, on the outer edge of the AAA+ ring (Fig. 6a). The small:large subdomain interface has been proposed to be relatively rigid (Carter et al., 2011) with most of the movement in the AAA+ ring occurring due to movements of the small subdomain relative to its own large subdomain. This may explain why the connectors can be ordered, as there is only minimal movement between small and neighbouring large subdomains.

The connector between AAA1 and AAA2 contains an extended region followed by an α-helix that packs against the H0 and H1 of AAA2L (Fig. 6b). In contrast the AAA2–3 connector is an entirely extended peptide (Fig. 6c). The AAA3–4 and AAA4–5 connectors, like the AAA1–2 connector, contain a region of extended polypeptide followed by an α-helix (Fig. 6d and e). In the AAA3–4 connector the α-helix contains the residues (K2721, D2725, E2726, E2727) involved in binding to Lis1 (Huang et al., 2012) and also possibly to the dynein neck (Roberts et al., 2012) which is at the very N-terminus of the dynein linker domain (Fig. 1b).

In the AAA4–5 connector (Fig. 6e) the first extended region wraps under the helices of the stalk, before coming up between the stalk and the buttress to join the H0 of AAA5. The region after H9 of AAA5 contains three extra helices that pack against, and extend, the AAA5 small subdomain (Fig. 6f). These extra helices are followed by a short extended region that connects to AAA6L.

Following the AAA6 domain, the *S. cerevisiae* dynein contains a C-terminal domain. The first part is an extended region of polypeptide and an α-helix (grey in Fig. 6g). This region does not pack between AAA6 and the neighbouring AAA+ domain (AAA1), but instead runs in the opposite direction back towards AAA5. The last part of the *S. cerevisiae* C-terminal domain is the C-terminal helix, which runs behind the AAA5 small subdomain (dark grey in Fig. 6g).

### 3.6 Evolutionary relationships between dyneins

The AAA+ family has been classified using two different approaches: classification based on structural features (Erzberger and Berger, 2006; Iyer et al., 2004) and classification based on sequence similarity (Ammelburg et al., 2006).

The morphological classification placed the dynein AAA+ domains in clade-7 (Erzberger and Berger, 2006), on the basis of a helix-2 insert, a PS-I insert and an additional helix in the small subdomain. The crystal structures of dynein showed that it does not have an extra helix between H5 and H6 (as in the case of the clade-7 proteins), but rather an extra helix after H8. In dynein, the small subdomain is formed from a compact five helix bundle. It sits in the conventional place with respect to the large subdomain and the sensor II arginine is present at the N-terminus of the third helix (H7) (Carter et al., 2011). By contrast, in the other clade-7 family members (BchI, MoxR and MCM) the H5-H6 insertion wraps around the back, or front, of the large subdomain and places the rest of the small subdomain including the sensor II arginine on the opposite side of the large subdomain.

Intriguingly, while the dynein AAA+ domains clearly lack the H5–H6 insertion, they may still be related to the clade-7 family. The last four helices in the dynein small subdomain (H6–H9) align very well with the four helices in MoxR (Fig. 4) that come after the H5/H6 insert. In the MoxR crystal structure (El Bakkouri et al., 2010) the small subdomain packs against the H5 helix from a neighbouring, symmetry related, small subdomain. This helix (shown as grey in Fig. 4) occupies the same position in the helical bundle as the regular H5 helix in dynein. The other clade-7 family members show a similar H5 to neighbouring small subdomain packing as observed in MoxR (Chen et al., 2010; Lundqvist et al., 2010; Slaymaker et al., 2013).

The clustering approach based on sequence suggested that the dynein AAA+ domains (AAA1–4) are only peripherally associated with the clade-7 proteins. Unlike the midasin domains, which all cluster together, the different dynein domains are quite separate from each other (Ammelburg et al., 2006). This is in contrast to our suggestion, based on the structural similarity between the dynein small subdomains, that all the dynein AAA+ domains are closely related to each other. The reason for this difference may be that the variety of inserts in the dynein large subdomains makes them appear more divergent in a BLAST based classification.

The proposed common ancestor for the dynein AAA+ domains raises the question of how the inserts in the large subdomain evolved. If dynein is related to other proteins in clade-7 (Iyer et al., 2004), then all dynein AAA+ domains originally contained a PS-I and H2 insert. All except AAA2 have lost their H2 insert and AAA6 has additionally lost the PS-I insert. Alternatively, the original dynein AAA+ domain could have contained only a PS-I insert (clade-5). This would mean that AAA2 gained a H2 insert and AAA6 lost the PS-I insert. Interestingly, the position of the H2 insert in dynein is nearer to the N-terminus of the helix than in PspF, NtrC and MCM. This might be consistent with it arising independently.

### 3.7 Differences between dyneins

The *S. cerevisiae* and *D. discoideum* dynein motor domains are only 30% identical at the sequence level. The structures of the individual motor domains are highly similar by contrast. The RMSD of the individual large subdomains is 1.95 Å. There are only four extra regions of sequence in the *D. discoideum* motor domain compared with that of *S. cerevisiae*. These differences are all in the small subdomains or connector segments and include: an extended H8 in AAA1, an insertion in the loop between H5 and H6 in AAA2 and an extra four helices in the connector between AAA5 and AAA6 that extends the small subdomain of AAA5. Finally, *D. discoideum*, like many other dyneins, also contains an additional C-terminal domain region containing six α-helices and a β-barrel domain that packs loosely against the small subdomain of AAA6. This part of the C-terminal domain is missing in some fungal cytoplasmic dyneins, but our alignment shows that it is actually present in other ones. The additional C-terminus region is missing in all Ascomycota (which include *S. cerevisiae*, as well as filamentous fungi such as Aspergillus), but are present in the Basidiomycota branch of the fungi. The extra region appears to be present in most other cytoplasmic dyneins and most axonemal dyneins. Interestingly, a small number of non-fungal dyneins also miss the C-terminal domain including cytoplasmic dynein from diatoms.

Plotting the sequence conservation of dynein suggests that there are very few surface conserved patches that are common to all dynein family members. These include the region around the AAA1 nucleotide binding pocket, the AAA2 inserts and regions on the linker. Given the diversity of functions of dynein, it is probable that different family members will show conserved surfaces that correspond to their specific functions. An example that has already been discussed (Schmidt et al., 2012), is the linker binding site on AAA5 which is found mainly in cytoplasmic dyneins. A related motif is found in IFT and inner-arm dyneins, but the site is completely missing in outer-arm dyneins. The analysis presented in this paper suggests that the site involving the PS-I insert on AAA4 is conserved in cytoplasmic dyneins, but is missing in other dyneins. It is highly possible that the inserts which have been the focus of this analysis paper will be used for specific interactions in different family members.

## 4 Conclusion

Although the motor domain of dynein follows the common architecture of AAA+ proteins in terms of overall fold and catalytic residues there are also interesting differences. The fact that all AAA+ domains are concatenated into a single polypeptide clearly distinguishes dynein from almost all other members of the large AAA+ family. The AAA+ domains of dynein have specialised with respect to each other. Specialisations include the presence of different combinations of H2 and PS-I inserts, different features within the inserts and extensions of the small subdomains of AAA4 and AAA5 resulting in the stalk and buttress, respectively.

## Figures and Tables

**Table 1:** Amino acid number boundaries of key features of the *S. cerevisiae* and *D. discoideum* motor domains.

		*S. cerevisiae*	*D. discoideum*
AAA1	Large	1776–1921	1950–2099
	Small	1922–2021	2100–2222
	H3–B4 insert	1874–1892	2053–2069
AAA1–2 connector		2022–2050	2223–2252
AAA2	Large	2051–2215	2253–2415
	Small	2216–2345	2416–2600
	H2 insert	2115–2128	2317–2331
	PS-I insert	2170–2190	2372–2391
AAA2–3 connector		2346–2394	2601–2650
AAA3	Large	2395–2558	2651–2815
	Small	2559–2680	2816–2937
	H2–B3 insert	2463–2475	2719–2732
	PS-I insert	2512–2529	2769–2786
AAA3–4 connector		2681–2734	2938–2990
AAA4	Large	2735–2916	2991–3174
	Small	2917–3355	3175–3624
	PS-I insert	2836–2885	3093–3141
AAA4–5 connector		3356–3388	3625–3654
AAA5	Large	3389–3518	3655–3785
	Small	3519–3710	3786–3978
	PS-I insert	3466–3493	3728–3762
AAA5–6 connector		3692–3771	3979–4117
AAA6	Large	3772–3897	4118–4238
	Small	3898–4015	4238–4363
C-terminal domain		4015–4092	4364–4730
